# Hepatocellular Carcinoma with Osteoclast-Like Giant Cells: Report of the Seventh Case in the Literature

**DOI:** 10.1155/2015/836105

**Published:** 2015-02-22

**Authors:** Lorenzo Dioscoridi, Damiano Bisogni, Giancarlo Freschi

**Affiliations:** Department of Surgery and Translational Medicine, University of Florence, Largo Brambilla 3, 50100 Florence, Italy

## Abstract

Hepatocellular carcinoma with osteoclast-like giant cells is extremely rare, and only six cases have been previously reported. Its histogenesis is at the moment controversial. The authors report a case of hepatocellular carcinoma with osteoclast-like giant cells found in a 74-year-old woman. The patient came with a dull pain in the right upper abdominal quadrants due to a liver neoplasm described at CT scan. A wedge resection of the fifth hepatic segment with appendectomy, omentectomy, and debulking of the major peritoneal implants was performed. Histologically, the diagnosis of hepatocellular carcinoma with high grade differentiation associated with giant osteoclast-like cells was done without any evidence of hepatitis or cirrhosis in the surrounding hepatic parenchyma. Immunohistochemistry was positive for CD10 and CD68 and in situ hybridization revealed the expression of receptor activator of nuclear factor-kappa B (RANK) in the giant cells and receptor activator of nuclear factor-kappa B ligand (RANKL) in the tumor cells.

## 1. Introduction

The first hepatocellular carcinoma with osteoclast-like giant cells was described in 1984 in a 54-year-old patient affected with cirrhosis [1]. Since then, five additional cases have been reported. Its histology is represented by the combination of hepatocellular carcinoma and giant cell tumor of the liver [2–4]. The histogenesis is controversial although extraskeletal giant cell tumors may occur in the soft tissue of many organs such as pancreas, thyroid gland, and liver. However, the characterization of the origin of giant cells in this kind of tumor remains incomplete. According to many reports, the osteoclast-like giant cells in tumors of organs other than the bone represent nonneoplastic histiocytes with stromal reaction, especially when this kind of cells was observed in a portion of a well-differentiated carcinoma [2, 5–7]. In the liver, several cases of hepatocellular carcinoma with formation of osteoclast-like giant cells have been reported as an extremely rare variant of HCC, and the hepatocyte-derived tumor cells were suggested to induce osteoclast-like giant cells [8–12]. This type of tumor is very aggressive: in all the cases, the patients died of the disease, more often, within one month after the diagnosis [4, 5, 7, 8, 13, 14]. Metastases have been observed in the vertebral bones and in the lung and are due to the sarcomatoid osteoclast-like component of the tumor [2, 15]. No evidence of a favourable effect of adjuvant or neoadjuvant chemotherapy is available at the moment and surgery seems to represent the treatment of choice [2, 5, 6, 13].

## 2. Case Report

A 74 year-old woman was admitted in our department with dull pain in the right upper quadrant associated with mild anemia (Hb 10.1). Oncological markers were in the normal range and the patient had no hepatopathies (HBV and HCV markers were negative).

**Figure 1:**
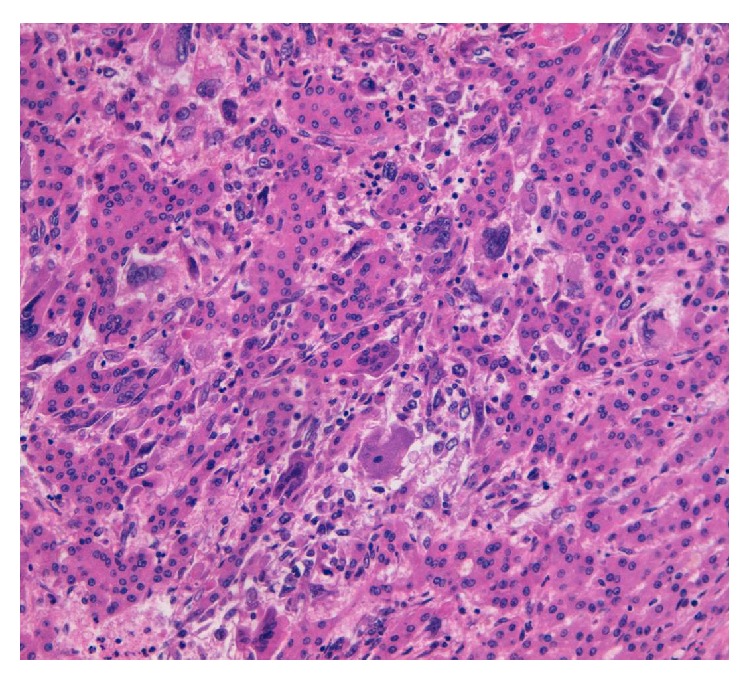
Hepatocellular carcinomatoid cells mixed with osteoclast-like giant cells (H&E, 20x).

A first line abdominal US showed a hepatic hypervascularized neoplasm of 10 × 7 centimeters. Thus, an upper abdominal CT scan was performed and the hepatic neoplasm was confirmed and shown to grow from a Riedel's segment towards the right iliac fossa with a close contiguity with ascending colon and caecum. A colonoscopy was also performed without any evidence of disease in the colon. The possibility of preoperative CT or US-guided biopsy was excluded on account of the hemorrhagic risk due to the CT hypervascularized appearance of the neoplasm. Therefore, the patient was scheduled for a wedge hepatic resection of the V-VI segments. However, at laparotomy, the understatement of the CT scan was evident. A wedge resection of the V-VI segments, cholecystectomy, appendectomy, omentectomy, and removal of few peritoneal neoplastic implants were performed in order to attain an apparently R0 resection. The histopatological exam evidenced a well-differentiated hepatocellular carcinoma with osteoclast-like cells that were well-represented especially in the peritoneal implants (see Figure 1). Immunohistochemistry revealed the expression of CD68, which is one of the markers of osteoclasts in osteoclast-like giant cells in the tumor. In situ hybridization revealed the expression of receptor activator of nuclear factor-kappa B (RANK) in the giant cells and receptor activator of nuclear factor-kappa B ligand (RANKL) in the tumor cells. The postoperative course was uneventful and the patient was discharged in the 8th postoperative day. Two months later, the patient was readmitted with severe anemia (Hb 7.3) and underwent a CT scan which showed a diffuse intraperitoneal carcinomatosis associated with huge ascites. At the palliative paracentesis the haemorrhagic character of ascites was seen and the patient died few weeks later (about four months after surgery) of multiple organ failure due to general hypovolemia and cardiac insufficiency. No other therapy was possible considering the fast progression of the disease.

## 3. Discussion

The present case of hepatocellular carcinoma with formation of osteoclast-like giant cells provided the occasion to review the literature concerning the topic and the previously reported cases. This type of tumor seems a very rare variant of HCC since only seven of them (including the present case) have been reported. If we consider the pathological and clinical characteristics of this neoplasm as they result from the published cases, few considerations can be done.

First of all, an almost uniform picture comes out from the analysis of the literature from clinical view-point: this tumor is very aggressive and imaging techniques usually underestimate the pathological situation [2, 13]. This is the reason why surgery is considered also useful for tumor staging. No staging scales are available because all the clinical cases described show very advanced forms with very poor prognosis.

Then, the patient had no previous hepatopathies and the liver parenchyma surrounding the tumor was completely normal at the histological examination. That condition has been already found in some other patients: in fact, two of the six previously reported cases had no hepatopathies [1–3, 10]. This condition seems more unusual for the hepatocellular carcinoma without osteoclast component that is generally associated with viral chronic hepatitis or cirrhosis.

Moreover, as well as for the previously reported cases, the oncological markers including alpha-fetoprotein were all in the normal range [10–12]. This may represent a further problem for screening and postoperative follow-up.

Finally, the natural history of the disease is, unfortunately, very short: the survival rate after surgery is few weeks despite the high grade of differentiation of the hepatocellular neoplastic cells [12, 13]. The osteoclast-like giant cells (recognizable by CD68 positivity at the immunohistochemistry) may induce greater aggressiveness and propensity to peritoneal colonization and metastasization: the neoplastic peritoneal implants are represented, in fact, by this kind of cells, not by the hepatocellular component [2, 9–12].

Several hypotheses have been reported regarding the histogenesis of sarcomatous components in carcinomas: (1) transdifferentiation or dedifferentiation from the original carcinoma cells, (2) biphasic differentiation from pluripotent stem cells, (3) metaplastic process of carcinoma, and (4) redifferentiation of immature multipotent carcinoma cells transformed from carcinoma cells [2, 4].

Osteoclast-like giant cells in carcinoma are generally considered reactive histiocytic cells rather than true malignant tumor cells but, in our case, they were responsible for peritoneal repetitions. About histogenesis of this type of cells in liver, they had a similar expression of almost all osteoclast markers of bone, including CD68, receptor activator of nuclear factor-kappa B (RANK), and RANK ligand (RANKL), suggesting that osteoclast-like cells in liver cancer had similar histogenesis of osteoclastogenesis in bone [2, 4, 12].

## 4. Conclusions

This hepatic combined tumor is of difficult diagnosis and therapy also on account of its rarity.

Its aggressive behaviour and poor prognosis might be positively conditioned by a better knowledge of its clinical history and of its relationship with concomitant hepatopathies, which does not seem to be so tight as for hepatocarcinoma tout-court.

Its histogenesis remains unclear and immunoistochemistry (CD10, CD68) is mandatory in order to demonstrate its osteoclast-like component.
